# From legislation to reality: Understanding gender-based violence in Tunisia

**DOI:** 10.1192/j.eurpsy.2024.1673

**Published:** 2024-08-27

**Authors:** K. Mahfoudh, S. Hamzaoui, S. Walha, D. Mezri, A. Ouertani, U. Ouali, A. Aissa, R. Jomli

**Affiliations:** Psychiatry “A”, Razi Hospital, Manouba, Tunisia

## Abstract

**Introduction:**

Tunisia marked a significant milestone in the fight against gender-based violence with the adoption of Organic Law No. 2017-58. This pioneering law in the region enhanced the protection of women and girls’ rights and introduced harsher penalties for perpetrators of sexist violence. However, the journey toward eradicating violence against women is complex and multifaceted.

**Objectives:**

Our aim is to explore how tunisian women perceive gender-based violence and their attitudes towards it.

**Methods:**

A cross sectional online survey designed using Google Forms and distributed on social media platforms (Facebook, Instagram) was conducted from August 30^th^ to September 25^th^ 2023. The questionnaire, presented in the tunisian dialect, included questions about personal experiences with violence, knowledge of gender-based violence laws as well as their perceptions and attitudes towards gender-based violence. The sample consisted of women from various regions of Tunisia.

**Results:**

In our study, we analyzed a sample comprising 110 tunisian women, with 46.4% falling within the 20 to 30 age bracket and 36.4% belonging to the 30 to 40 age range. Half of the survey participants were unmarried, and the majority of them (97.3%) had attained a university-level education.

Our research revealed that 45.5% of the surveyed women reported instances of gender-based violence in Tunisia. However, only a minority of these individuals (22.2%) initiated legal proceedings, primarily citing a lack of confidence in the judicial system and fear of potential reprisals as their reasons.

A majority of the participating women expressed deep concern regarding the issue of violence against women in Tunisia.

When asked about their perceptions of the most prevalent types of violence in Tunisia, 76.36% believed that psychological violence was the most common, followed by sexual violence (21.3%). Economic and physical violence were perceived as less frequent (9.9%; 12.6%). These women attributed the primary factors contributing to violence against women in Tunisia to cultural norms and laws that they considered inadequately stringent. Indeed, 83.3% of them believed that the current legislation was not stringent enough to deter potential perpetrators, and 37.3% indicated that they were unaware of the existing legal framework.

**Conclusions:**

Despite legislative advancements, gender-based violence remains a pressing concern in Tunisia. These findings underscore the importance of increasing awareness about available resources for victims, educating individuals about women’s rights and mental health, and building trust in the judicial system.

**Disclosure of Interest:**

None Declared

